# Legends of Allergy & Immunology: A. Wesley Burks: Advocate for Children's Health Through Scientific Discovery

**DOI:** 10.1111/all.70317

**Published:** 2026-03-27

**Authors:** M. Cecilia Berin, Stacie M. Jones, Edwin H. Kim, Hugh A. Sampson

**Affiliations:** ^1^ Department of Medicine Northwestern University Feinberg School of Medicine Chicago Illinois USA; ^2^ Department of Pediatrics University of Arkansas for Medical Sciences and Arkansas Children's Hospital Little Rock Arkansas USA; ^3^ Department of Medicine and Pediatrics University of North Carolina School of Medicine Chapel Hill North Carolina USA; ^4^ Jaffe Food Allergy Institute, Icahn School of Medicine at Mount Sinai New York New York USA

**Major Contributions to Food Allergy**

Dr. Burks and colleagues:

- Identified and characterized the major allergens of peanut, providing the foundation for diagnostics and new therapies (1991–1999).
- Performed the first oral immunotherapy trials of peanut oral immunotherapy in the United States (2009), culminating in the first FDA‐approved treatment for food allergy (Palforzia) in 2020. Performed the first clinical trial of sublingual immunotherapy for the treatment of peanut allergy (2011).
- First description of sustained unresponsiveness after egg oral immunotherapy (2012).
- Led the IMPACT clinical trial that identified an early window of opportunity for lasting remission in response to peanut oral immunotherapy (2022).

The field of food allergy in 2026 is thriving, with therapeutic options for patients, a robust treatment pipeline, and active participation from basic scientists and industry partners. This is a stark contrast to the state of the field when Dr. Wesley Burks began and can be attributed in large part to his work and leadership.

Dr. Burks (photo in Figure 1) began his career at the University of Arkansas for Medical Sciences (UAMS), after a fellowship in Allergy/Immunology at Duke University, where his interest in food allergy was sparked during work with new faculty member Dr. Hugh Sampson, a collaboration and friendship that has now spanned 40 years. At UAMS, Dr. Burks set out to identify the molecular basis of peanut allergy. Working with colleagues Drs. Rick Helm and Gary Bannon, he identified the major peanut allergens Ara h 1–3. In a seminal work in 1995, their team produced recombinant Ara h 1 and modified the protein to prevent IgE binding [1]. These hypoallergenic peanut proteins were proposed to be a safer approach to allergen immunotherapy, a concept tested in the first clinical trial run by the Consortium for Food Allergy Research (CoFAR), of which Dr. Burks was an inaugural member. Although the modifications made were not sufficient to prevent allergic reactions in all participants, the investigation of molecular determinants of peanut allergy rests on his formative work.

**FIGURE 1:**
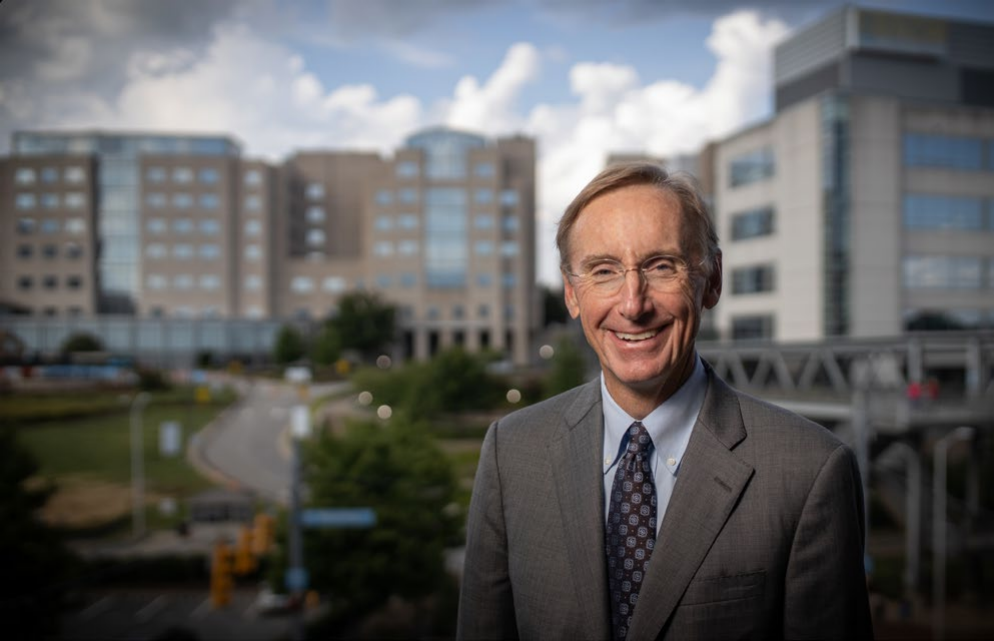
Dr. A. Wesley Burks, MD.

In 2003, Dr. Burks moved back to Duke University and began to investigate whether the immune phenomenon of oral tolerance could be harnessed to treat food allergy. He established a robust clinical program paired with translational immunology to investigate the use of oral immunotherapy (OIT) for the treatment of food allergy. In partnership with Dr. Stacie Jones at UAMS, they laid the groundwork for OIT as a treatment for food allergy [2, 3]. This work fed into the first multi‐center randomized placebo‐controlled trial of egg OIT revealing immune regulation and clinical efficacy and the first demonstration of sustained unresponsiveness [4]. These trials paved the way for the development, testing [5], and approval of the first commercially available OIT product for treatment of the peanut allergy. For the first time, allergists could offer their food‐allergic patients a treatment path that could protect them from allergic reactions. During this time, Dr. Burks moved to the University of North Carolina as Chair of Pediatrics (2011), followed by Dean of the School of Medicine (2015), and subsequently CEO of UNC Health Care (2019). He is currently (since 2025) Board Chair and Administrator of the first freestanding Children's Hospital in North Carolina. The impact of his dedication to children's health is felt far beyond the field of allergy and immunology. Despite these major administrative responsibilities, Dr. Burks has maintained a vibrant collaborative research program. Dr. Burks has been instrumental in continuing investigations to improve OIT and has led efforts to identify the early window of opportunity for establishing long‐lasting remission by OIT [6]. He has also led the promising development of sublingual immunotherapy for the treatment of food allergy with Dr. Edwin Kim, and together with Dr. Michael Kulis, continues to advance new immunotherapy approaches and identify mechanisms of treatment response.

Dr. Burks' impact on the field of food allergy will endure far beyond his own career. His mentorship has played a key role in the career development of innumerable leaders in the food allergy field. His mentees have a consistent message: that Dr. Burks gave them guidance and room to learn to trust their own best judgment using the guiding principle of “do the right thing”. That mentoring philosophy is now being handed down to the next generation of physicians and scientists. His impact on the next generation is much broader than his direct mentees: through his tireless service to the allergy community through National Institutes of Health study section service as reviewer and Chair, and his leadership as President of the American Academy of Allergy, Asthma, and Immunology, he has encouraged and supported countless physicians and scientists to dedicate their careers to the field of allergy. Ultimately, it is through his humanism and deep personal commitment to improving the lives and health of children and families that inspire those around him the most. The future of the field is bright, promising improved lives for those affected by food allergy, in no small part due to the remarkable innovation, leadership, and humanity of Dr. Wesley Burks.

## Funding

The authors have nothing to report.

## Conflicts of Interest

The authors declare no conflicts of interest.

## Data Availability

Data sharing not applicable to this article as no datasets were generated or analyzed during the current study.
